# Use of Personal Protective Equipment among Building Construction Workers in Kampala, Uganda

**DOI:** 10.1155/2017/7930589

**Published:** 2017-10-23

**Authors:** Jonathan Izudi, Viola Ninsiima, John Bosco Alege

**Affiliations:** ^1^Department of Community Health, Faculty of Medicine, Mbarara University of Science and Technology, Mbarara, Uganda; ^2^Institute of Public Health and Management, International Health Sciences University, Kampala, Uganda

## Abstract

**Background:**

270 million workplace accidents occur annually. In Uganda, Kampala district has the highest workplace injury and fatality rates. However, information on personal protective equipment (PPE)—hand gloves, hardhats, overalls, safety boots, earplugs, safety harness with lanyard, and face shields—utilization among building construction workers remains scarce. We assessed PPE utilization and determinants among building construction workers in Kampala, Uganda.

**Methods:**

This cross-sectional study involved 385 respondents. Data collected by structured questionnaire was double-entered in EpiData and analyzed in STATA at 5% significance level. Independent determinants of PPE use were established by a stepwise backward logistic regression analysis.

**Results:**

305 (79.2%) respondents were males, 290 (75.3%) were 18–30 years, 285 (74.0%) completed secondary education, and 197 (51.2%) were temporary employees. 60 (15.6%) respondents used PPE. Female sex (adjusted odds ratio (AOR) = 6.64; 95% CI: 1.55–28.46; *P* = 0.011), temporary (AOR = 0.05; 95% CI: 0.01–0.27; *P* < 0.001) and casual (AOR = 0.01; 95% CI: 0.001–0.071; *P* < 0.001) employment, and previous knowledge of safety measures (AOR = 100.72; 95% CI: 26.00–390.16; *P* < 0.001) were associated with PPE use.

**Conclusion:**

PPE use was low in Kampala, Uganda. Building construction companies should implement measures of the Uganda Occupational Health and Safety Act.

## 1. Introduction

Workplace injuries are a leading cause of substantial disabilities globally [1]. According to the International Labor Organization, 270 million workplace accidents occur annually [2]. Construction employees have 1 in 300 chances of dying while at workplace and the likelihood of becoming handicapped by injury is far greater than in other industries [3]. In particular, falls top the list of building construction site accidents and, in most cases, it results in considerable number of deaths. Falls also singly account for a large proportion of the total number of accidents and fatalities among construction workers [4]. In general, construction site accidents result in sizeable pain, suffering, lowered productivity, reduced quality of life, and loss of time. On average, building construction accidents that cause permanent disability reduce worker capacity by 37% [5]. Surprisingly, in most developing countries, health and safety considerations at construction projects are not a priority, and the use of safety measures is considered a burden.

In Uganda, the Occupational Health and Safety (OHS) Act Number 9 of 2006 requires all employers to provide a safe working environment besides safety measures to employees and employees to comply with safety measures in place [6]. However, nonemployees like casual workers (working without contracts or formal appointment letters) are not protected by this law. The absence of contracts or formal appointments means they cannot sue employers for compensation in the event of injury, accident, or death. The stated Act also entails all enterprises to comply with OHS standards [6, 7].

Nevertheless, compliance to these safety measures remains noticeably low. For example, in November 2015, the Ministry of Gender, Labor and Social Development closed 12 enterprises in Kampala district due to nonimplementation and noncompliance with safety rules [7]: one risk factor for fatal accidents, injuries, and deaths [8, 9].

Between 2001 and 2005, collapse of building elements and improper use of machines caused substantial accidents at building construction sites in Uganda. Notably, hit by objects and falls were a major cause of the accidents [5]. Rates of deaths and serious injuries in Kampala district, Uganda, are the highest in the country, with the construction sector rated as the third most hazardous place. Statistics indicate that the construction sector accounts for 9% of all occupational fatalities and 18% of all occupational injuries [6]. In 2014, the injury rate for Kampala district was 3,797 per 100,000 workers and the fatality rate was 84 per 100,000 workers mainly from mechanical hazards (struck by machines, vehicles, hand tools, and cutting edges), hit by falling objects, and falls from heights [10]. In the building construction sector, good compliance to use of personal protective equipment (PPE) like gloves, hardhats, overalls, safety boots, ear plugs, face shield, and safety harness with lanyard alongside other safety measures is critical in reducing the incidences of injuries and deaths [11]. For instance, face shields (such as nose masks, safety glasses, and safety googles) protect the face and eye, work shoes (gumboots) with slip and puncture resistant soles protect the foot, hardhats protect the head and face, earplugs (earmuffs) protect the ears, and hand gloves of the right size (preferably heavy-duty rubber gloves) protect the hand [12].

Despite numerous current building construction projects and high injury and fatality rate in Kampala district, Uganda, information on use of PPE and its determinants among building construction workers is lacking. This study consequently investigated the use of PPE and its determinants among building construction workers in Kampala, Uganda.

## 2. Methods and Materials

This study was conducted at one site (the construction of the central teaching facilities and refurbishment of old laboratories, Makerere University, Kampala, Uganda), in particular, among building construction workers of Excel Construction Limited (ECL). ECL is a leading player in the Ugandan Construction industry headquartered in Kampala. ECL was formed in 1992 as a joint venture between Muljibhai Madhvani Company and Gomba Construction Limited and underwent training, auditing, and certification in the implementation of ISO 9001:2008 quality management systems in the construction industry [13].

We used an analytical cross-sectional design to describe and analyze factors associated with use of PPE among building construction workers. 385 respondents were included in this study using Kish and Leslie's formula [14], within 95% confidence limits, 5% precision, and 50% hypothetical proportion of PPE use. In our setting, all construction workers (permanent, temporary, and casual workers) had a unique identification code that was used for sampling. An independent person performed a simple random sampling using these unique identification codes to generate a list of random numbers with a computer.

The numbers were then used to identify, approach, and obtain informed consent from potential respondents before interviewing. Interviews were conducted by trained and supervised Research Assistants onsite (but in a private, quiet, and convenient room away from the rest of the construction workers) from Monday to Friday between 9.00 am and 3.00 pm. Two Research Assistants conducted the interviews using a structured questionnaire between August and October 2016 in the local language (Luganda). Before the interviews, the structured questionnaire was forward and backward translated by two independent local translators (one translated from English to Luganda, and the other back translated the Luganda version to English). Afterwards, the two translators met, harmonized their translations, and developed the final questionnaire. On average, three participants were interviewed per day by each Research Assistant and none of the respondents declined the interview. At the end of each day of data collection, a Research Team Leader reviewed all completed questionnaires for completeness and accuracy.

Collected data was double-entered in EpiData version 3.1 (EpiData Association, Odense, Denmark) [15] and exported to STATA Version 12 (StataCorp, College Station, TX, USA) for univariate, bivariate, and multivariate analysis at 5% significance level. The outcome variable, use of PPE, was computed as the percentage of respondents that had worn a pair of hand gloves, hardhat, overall and safety boots, and either an earplug or a face shield or safety harness with lanyard. Participants were asked if they had worn any of the PPE during work in the day or within 2 days of work at the building construction site.

The independent factors were individual (awareness on occupational hazards and risks, age in years, level of education, attitude towards use of PPE, and prior safety knowledge), level of supervision (supervision frequency and onsite safety checks), type of employment (permanent, temporary, or casual worker), and industrial (availability of work safety policies/guidelines and safety training) factors.

In univariate analysis, we computed frequencies and percentages for categorical variables, means with standard deviations, and medians with interquartile ranges (IQR) for numerical variables. In bivariate analysis, the Chi-squared test was used to analyze the association between categorical independent variables and the outcome variable when all the cell counts were equal to five or above, and the Fisher exact test was used when any of the cell counts was less than five. Conversely, associations between numerical independent variables and the outcome variable were assessed by Student's *t*-test. A univariable binary logistic regression analysis was conducted for significant variables at bivariate analysis to determine strengths of associations, and the results were stated in unadjusted odds ratios (UOR). For significant variables at univariable analysis, a final multivariable binary logistic regression analysis was performed to determine independent determinants of PPE use. The results were stated in adjusted odds ratios (AOR). The final multivariate logistic regression analysis model showed that the data fitted fairly well (log-likelihood = −74.67, pseudo *R*-squared = 0.78, and *P* < 0.0001). In the analyses, all odds ratios were stated with corresponding 95% confidence intervals (CI) and *P* values.

This study was approved by the Research Ethics Committee of International Health Sciences University, Institute of Public Health and Management, Kampala, Uganda. In addition, we obtained a written informed consent from all study participants.

## 3. Results

### 3.1. Sociodemographic Characteristics of Respondents and Use of PPE

Of the 385 respondents, 305 (79.2%) were males, 290 (75.3%) were aged 18–30 years, 285 (74.0%) completed secondary education, and 197 (51.2%) worked on temporary basis (Table 1). Overall, 60 (15.6%) out of 385 respondents used PPE.

### 3.2. Factors Associated with Use of PPE among Building Construction Workers

In bivariate analysis, gender (*P* < 0.001), form of employment (*P* < 0.001), prior knowledge of safety measures (*P* < 0.001), number of safety training times (*P* = 0.020), and number of Continuous Professional Education (CPE) sessions attended per month (*P* = 0.002) were statistically significantly associated with use of PPE. However, the respondents' age group, level of education, receipt of work safety messages, provision of safety guidelines, and work safety policy were not statistically significantly associated with use of PPE (Supplementary Material S1 available online at https://doi.org/10.1155/2017/7930589).

In univariable analysis, female respondents used PPE more than male respondents. Respondents aged 31–45 years and 46–60 years also used PPE more than those aged 18–30 years.

Respondents employed as temporary and casual workers were less likely to use PPE compared to permanently employed workers (Table 2). Respondents that had prior knowledge on safety measures compared to those that had no knowledge were more likely to use PPE. In contrast to 1-2 safety training times per month, three or more safety training times per month was associated with less likelihood of using PPE. Also, respondents that had attended three and more Continuous Professional Education (CPE) sessions per month compared to those that attended 1-2 CPE sessions per month were more likely to use PPE (Table 2).

In multivariate analysis (adjusted for all statistically significant factors at univariable analysis), female respondents and respondents with past knowledge of safety measures were more likely to use PPE. However, respondents employed as temporary and casual workers compared to permanent workers were less likely to use PPE (Table 2). Conversely, use of PPE was similar by age group (31–45 years, 46–60 years, and 18–30 years), ever attending three or more safety training times per month, and ever attending three or more Continuous Professional Education (CPE) sessions per month (Table 2).

## 4. Discussion

This study found low use of PPE (15.6%) among building construction workers in Kampala, Uganda, compared to 100% as recommended in the Uganda OHS Act [6, 7]. This was not surprising because in Kampala district, Uganda, only 1,175 out of several enterprises comply with the OHS Act [7]. The low use of PPE confirms previous evidence that showed high occupational injuries and fatal accidents in the construction industry [5, 10]. However, the use of PPE in this study was lower than 27.8% reported among auto-technicians in Uyo, Nigeria [16]. Our result therefore calls for urgent strict enforcement of the use of PPE at building construction sites in Kampala, Uganda.

Our study indicates that prior knowledge of safety measures increased use of PPE. Essentially, ignorance and inadequate health and safety information are dual factors that contribute substantially to poor safety practices at construction sites. Earlier, lack of safety training affected use of PPE [17]. In Kenya, lack of health and safety training among construction workers reduced used of PPE [3]. In Nigeria, lack of general knowledge on workplace safety among auto-technicians also reduced use of PPE [16].

Also previously in Kampala, Uganda, the employment of incompetent workers was associated with increased accidents at construction industries [18]. Our result therefore confirms the evidence that training employees in safety measures is vital in increasing their knowledge, competence, and use of safety measures at the workplace [17].

This study found permanently employed building construction workers used PPE more often than casual and temporarily employed workers. Past research evidence in Uganda indicates that the greatest sufferers of accidents are workers at building construction sites followed by carpenters and plant operators [5]. This trend implies that rates of accidents depend on the type of work that in turn is influenced by differences in skills, knowledge, and competence. Generally, casual and temporary employees perform much of the tasks at building construction sites despite inadequate safety knowledge [5]. The low level of skills and knowledge of safety measures among casual and temporary employees probably accounted for the low use of PPE. In our setting, permanently employed workers had high level of formal training (mostly tertiary and university education) in contrast to casual and temporary workers that formed the bulk of laborers. This implies that permanently employed workers had good knowledge of safety measures compared to casual and temporary employees. It is therefore not surprising that permanently employed workers used PPE more often than casual and temporary workers.

We found female workers used PPE more often than male workers. There are no published studies to explain this finding. However, the building construction industry is largely dominated by men globally. The only supporting evidence comes from the International Labor Organization report that stated men are less likely to adopt preventive and protective measures at workplace compared to women [19]. It is therefore not surprising that female workers had increased use of PPE than male workers.

Based on common understanding, women naturally fear harmful objects and risky tasks more than men, and culturally, in Uganda, women are expected to be less involved in construction work than men. These perceptions therefore shape their attitudes and behavior at work resulting into less involvement in tasks that pose harm. It might therefore be this fear factor that contributed to raised level of precaution and hence increased use of safety measures like PPE.

By and large, our findings recognize the need to improve the work environment as a whole and the impossibility of implementing PPE use without following the Uganda OHS Act. In the real world, people act because of different motivations or by habits without explicit motives. Nevertheless, motives in construction industry primarily do not follow the law in most countries (including Uganda). This implies that social factors, culture, education, and availability of good PPE among others are important.

## 5. Study Limitations

For the first time in Uganda, the present study highlighted use of PPE among building construction workers and sets a platform for prospective research in improving adherence to Occupational Health and Safety. Besides, it contributes to the existing body of knowledge on health and safety standards in the growing building construction industry in Uganda. However, this study has limitations that should be considered in the interpretation. We used a cross-sectional design where use of PPE was assessed once.

An observational study on site for longer periods would have been a strength but requires many resources. Besides, there is a possibility of reporting bias during interviews and our questionnaire was invalidated. In addition, our findings are limited to building construction sites in Kampala, Uganda, and the application of these results in other building construction sites in Uganda or other industries (processing or manufacturing among others) may not be valid.

## 6. Conclusion and Recommendation

Our study indicates low use of PPE among building construction workers in Kampala, Uganda. Female sex, permanent employment, and knowledge of safety measures were associated with increased use of PPE. To prevent injuries and deaths at building construction sites, companies should implement the use of PPE.

## Supplementary Material

Factor associated with use of PPE at bivariate analysis.

## Figures and Tables

**Table 1:** Sociodemographic characteristics.

Variables	Number (Total = 385)	Percentage (Total = 100%)
Gender		
Male	305	79.2
Female	80	20.8
Age group (years)		
18–30	290	75.3
31–45	80	20.8
46–60	15	3.9
Educational level		
Primary	40	10.4
Secondary	285	74.0
Tertiary	60	15.6
Form of employment		
Permanent worker	39	10.1
Temporary worker	197	51.2
Casual worker	149	38.7

**Table 2:** Factors associated with use of PPE among building construction workers in Kampala, Uganda.

Variable	Used PPE?		Univariable logistic regression analysis		Multivariable logistic regression analysis	
	No (*n* = 325)	Yes (*n* = 60)	UOR (95% CI)	*P* value	AOR (95% CI)	*P* value
Gender						
Male	270 (88.5)	35 (11.5)	1		1	
Female	55 (68.8)	25 (31.2)	3.51 (1.94–6.32)	<0.001	6.64 (1.55–28.46)	0.011
Age in years						
18–30	250 (80.2)	40 (13.8)	1		1	
31–45	65 (81.3)	15 (18.8)	1.44 (0.75–2.77)	0.272	1.62 (0.55–4.73)	0.379
46–60	10 (66.7)	5 (33.3)	3.13 (1.02–9.62)	0.047	5.89 (0.67–51.65)	0.109
Form of employment						
Permanent worker	24 (16.5)	15 (38.5)	1		1	
Temporary worker	162 (82.2)	35 (17.8)	0.35 (0.16–0.73)	0.005	0.05 (0.01–0.27)	<0.001
Casual worker	139 (93.3)	10 (6.7)	0.12 (0.05–0.29)	<0.001	0.01 (0.001–0.071)	<0.001
Past knowledge of safety measures						
No	305 (95.3)	15 (4.7)	1		1	
Yes	20 (30.8)	45 (69.2)	45.80 (21.8–95.8)	<0.001	100.72 (26.00–390.16)	<0.001
Safety training per month						
1-2	24 (70.6)	10 (29.4)	1		1	
3 and over	301 (85.5)	50 (14.3)	0.40 (0.18–0.88)	0.024	1.10 (0.18–6.61)	0.916
CPEs per month						
1-2	150 (90.9)	15 (9.1)	1		1	
3 and over	175 (79.6)	45 (20.6)	2.57 (1.38–4.89)	0.003	0.53 (0.12–2.38)	0.408

*Note*. Percentages are calculated as row percentages (*n*/*N*); AOR: adjusted odds ratio; UOR: unadjusted odds ratio; *P* < 5% is statistically significant. Multivariable logistic regression analysis involved all variables with *P* < 5% at univariable analysis; CPE: Continuous Professional Education.

## Abbreviations

AOR: Adjusted odds ratio
CPE: Continuous Professional Education
UOR: Unadjusted odds ratio
PPE: Personal protection equipment
OHS: Occupational Health and Safety.
