# Supplementary material for: Use of Personal Protective Equipment among Building Construction Workers in Kampala, Uganda
Source: J Environ Public Health. 2017 Oct 23;2017:7930589. doi: 10.1155/2017/7930589 (PMC5672632; doi:10.1155/2017/7930589)
Supplement: Supplementary file 1 — Factor associated with use of PPE at bivariate analysis. [file 7930589.f1.pdf]

Supplementary Material S1: Use of PPE by building construction workers in Kampala district, Uganda

	Used PPE			
**Variable**	**No**	**Yes**	**Total**	P-value
Overall use of PPE	325 (84.4)	60 (15.6)	385	P<0.0001
Gender				P<0.001
Male	270 (88.5)	35 (11.5)	305	
Female	55 (68.8)	25 (31.2)	80	
Age in years				
18-30	250 (80.2)	40 (13.8)	290	P=0.086
31-45	65 (81.3)	15 (18.8)	80	
46-60	10 (66.7)	5 (33.3)	15	
Educational level				P=0.088
Primary	35 (87.5)	5 (12.5)	40	
Secondary	245 (86.0)	40 (14.0)	285	
Tertiary	45 (75.0)	15 (25.0)	60	
Form of employment				P<0.001
Permanent	24 (16.5)	15 (38.5)	39	
Temporary	162 (82.2)	35 (17.8)	197	
Casual	139 (93.3)	10 (6.7)	149	
Ever heard of safety measures				P=0.334
Yes	320 (84.2)	60 (15.8)	380	
No	5 (100.0)	0 (0.0)	5	
Prior knowledge of safety measures				P<0.001
No	305 (95.3)	15 (4.7)	320	
Yes	20 (30.8)	45 (69.2)	65	
Provided with work safety guidelines				P=0.614
Yes	174 (85.3)	30 (14.7)	204	
No	151 (83.4)	30 (16.6)	181	
Provided with work safety policy				P=0.570
Yes	290 (84.1)	55 (15.9)	345	
No	35 (87.5)	5 (12.5)	40	
Number of safety trainings per month				P=0.020
1-2	24 (70.6)	10(29.4)	34	
3 and over	301 (85.5)	50 (14.3)	351	
Number of continuous professional educations per month				P=0.002
1-2	150 (90.9)	15 (9.1)	165	
3 and over	175 (79.6)	45 (20.6)	220	
